# Accelerated Photo‐Induced Degradation of Benzidine‐*p*‐Aminothiophenolate Immobilized at Light‐Enhancing TiO_2_ Nanotube Electrodes

**DOI:** 10.1002/chem.201902963

**Published:** 2019-11-18

**Authors:** Christine Joy Querebillo, Ibrahim Halil Öner, Peter Hildebrandt, Khoa Hoang Ly, Inez M. Weidinger

**Affiliations:** ^1^ Professur für Elektrochemie Technische Universität Dresden 01062 Dresden Germany; ^2^ Institut für Chemie Technische Universität Berlin Straße des 17. Juni 135 10623 Berlin Germany; ^3^ School of Analytical Sciences Adlershof Unter den Linden 6 10099 Berlin Germany

**Keywords:** azo compounds, nanotubes, photodegradation, photonic activity, resonance Raman spectroscopy

## Abstract

Herein, the enhanced visible‐light‐induced degradation of the azo‐dye benzidine‐*p*‐aminothiophenolate immobilized on TiO_2_ nanotube electrodes is reported. Exploiting the reported photonic properties of the TiO_2_ support and the strong electronic absorption of the dye allowed for employing surface‐enhanced resonance Raman spectroscopy at 413 nm to simultaneously trigger the photoreaction and follow the time‐dependent decay process. Degradation rate constants of up to 25 s^−1^ were observed, which stand among the highest reported values for laser‐induced degradation of immobilized dyes on photonically active supports. Contrast experiments with two differently light‐enhancing TiO_2_ nanotube electrodes establish the direct correlation of the material's optical response, that is, electromagnetic field enhancement, on the interfacial photocatalytic reaction.

## Graphical abstract

**Enhanced degradation** rates of benzidine‐*p*‐aminothiophenolate on TiO_2_ nanotube electrodes were derived. The photonic properties of the TiO_2_ support and the strong electronic absorption of the dye allowed for employing surface‐enhanced resonance Raman spectroscopy to simultaneously trigger the photoreaction and follow the time‐dependent decay process.

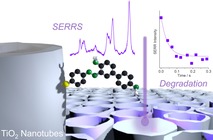

## Introduction

Titanium dioxide, TiO_2_ or titania, is considered one of the best‐studied semiconductors to date.^1^ Due to its extraordinary properties such as the high stability to chemicals and corrosion as well as the low toxicity and biocompatibility, the low‐cost material TiO_2_ has become indispensable for many technological applications. As a semiconductor, TiO_2_ exhibits a wide band gap that can be resonantly excited using soft UV light. The highly reducing and oxidizing potential of its conduction and valence band (CB and VB), respectively, have promoted the broad applicability of TiO_2_ for hosting a range of light‐driven reactions at its interface.^1^, ^2^

TiO_2_ has been intensely investigated with respect to photocatalytic remediation of environmental pollutants.^3^ Upon UV‐light absorption, mobile charge carriers are generated in the TiO_2_ that can react at the interface to produce highly reactive molecular species capable of decomposing toxic organic molecules into nontoxic products.^3^, ^4^ Visible light‐induced degradation of pollutants at a TiO_2_ interface proceeds without the involvement of charge carriers from TiO_2_.^5^ Here, light absorption by molecular pollutants at the interface is followed by excited‐state electron transfer into acceptor states in the TiO_2_, unlocking several pathways for the subsequent degradation of the absorbing species.

On the quest of improving the capability of TiO_2_ to mediate light‐induced degradations, particularly, the ability of periodic TiO_2_ nanostructures to form photonic crystals has been recognized as a versatile tool to enhance photocatalysis at TiO_2_ interfaces without the involvement of auxiliary atoms.^6^, ^7^ TiO_2_ structures with inverse‐opal and nanotubular geometry have been shown to afford enhanced photocatalytic degradation of organic dyes upon visible‐light irradiation.^8^, ^9^, ^10^, ^11^ The origin of the enhancement is typically ascribed to the materials’ photonic properties resulting from a periodic modification of the refractive index at the scale of the incident wavelengths.^9^, ^12^ In this way, light energy is “trapped” within the well‐defined nanostructures forming photonic lattices and allowing for highly enhanced photon interaction (i.e., absorption) with the material and associated molecules. Moreover, the formation of nanostructures intrinsically affords greater active surface area. Another advantage of such solid TiO_2_ arrays is the ability to be readily electro‐contacted and employed as an electrode to facilitate desired interfacial charge‐transfer reactions by applying an external bias. Furthermore, such arrays can be readily incorporated in technologically relevant photocatalytic flow cells and swiftly recycled through annealing, providing an edge compared with typically employed nanoparticle suspensions. These qualities combined make ordered nanostructured TiO_2_ highly interesting for applications in photocatalytic conversion of environmental pollutants.

Recently, we reported highly enhanced localized electromagnetic fields near the solid–liquid interface within anodized TiO_2_ nanotubular structures at 413 nm laser excitation.^13^ In the present work, we aim at investigating their contribution to photocatalytic degradation of organic dyes at anodized TiO_2_ nanotubes (TiO_2_‐NTs) interfaces. As photo‐active target, the azo‐dye benzidine‐*p*‐aminothiophenol (BD‐PATP), a known environmental pollutant and reported human carcinogen, was chosen as a model dye.^14^ Its degradation pathway is well understood^4^, ^15^ and resembles those of other dyes on TiO_2_ 15 allowing for generalizing the findings to other environmentally relevant compounds. Exploiting the strong electronic absorption of the dye, time‐resolved surface‐enhanced resonance Raman was used to concomitantly trigger and monitor the degradation of the azo dye, which has been prior immobilized on the TiO_2_ interface. In contrast to typical performance‐based studies, this study focuses on the interfacial degradation reaction and the influence of enhanced electromagnetic fields on accelerating such reactions to shine light on relevant optical requirements for highly active photoactive supports.

## Results and Discussion

**Figure 1:**
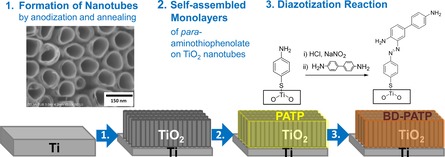
Fabrication of dye‐modified TiO_2_ nanotube electrodes. A scheme showing the general procedure for adsorbing the sample dye benzidine‐*p*‐aminothiophenol/ate (BD‐PATP) on anodized TiO_2_‐NT electrodes.

The preparation of the dye‐modified electrodes followed a three‐step procedure as shown in Figure 1. Two different types of TiO_2_‐NT electrodes were prepared following a recently published protocol.^13^ Briefly, TiO_2_ was obtained by anodization of Ti foils in fluoride‐rich media. Subsequent annealing at different temperatures afforded the different optical properties (Figure 1, step 1). We showed previously that applying an annealing temperature of 300 and 475 °C results in TiO_2_‐NT electrodes with lower and higher pronounced localized electromagnetic fields at the TiO_2_‐NT–liquid interface, respectively.^13^ In the present study, such electrodes annealed at 300 and 475 °C will be referred to as TiO_2_‐NT|low and TiO_2_‐NT|high, respectively.

**Figure 2:**
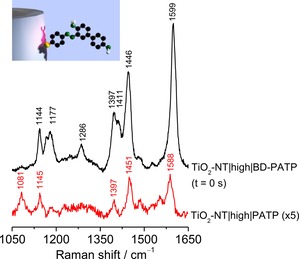
SER and SERR spectra of PATP and BD‐PATP on TiO_2_‐NT|high (black and red trace, respectively) showing the successful formation of the azo‐dye BD‐PATP. Characteristic peaks for each adsorbate are labelled. Conditions: 10 s, average of 10 spectra; laser excitation: 1 mW at 413 nm laser excitation in water.

In contrast to many studies reporting photocatalytic degradation on TiO_2_ materials, the target dye BD‐PATP was chemically attached to the TiO_2_ surface. To achieve this, first a monolayer of *para*‐aminothiophenol (PATP) was adsorbed (Figure 1, step 2) on the TiO_2_‐NT surface by incubation of the electrode in a 2 mm ethanolic solution. PATP‐modified electrodes are denoted as TiO_2_‐NT|low|PATP and TiO_2_‐NT|high|PATP. The presence of PATP on TiO_2_‐NT|high after thorough rinsing with abundant ethanol was verified by surface‐enhanced Raman (SER) spectroscopy (Figure 2). BD‐PATP dye formation was achieved by inducing an azo‐coupling reaction following reported procedures (Figure 1, step 3).^16^

BD‐PATP exhibits a strong electronic absorption due to extended delocalization of π electrons affording strong resonance Raman (RR) signals of the dye at 413 nm laser excitation within the photonic band gap of the anodized TiO_2_‐NTs (Figure 2).^13^, ^17^ Formation of BD‐PATP was therefore readily confirmed by surface‐enhanced resonance Raman (SERR) spectroscopy. The resulting dye‐modified electrodes are denoted as TiO_2_‐NT|low|BD‐PATP and TiO_2_‐NT|high|BD‐PATP. Importantly, the significantly higher signal intensity of the BD‐PATP over PATP made it possible to perform time‐dependent SERR studies.

**Figure 3:**
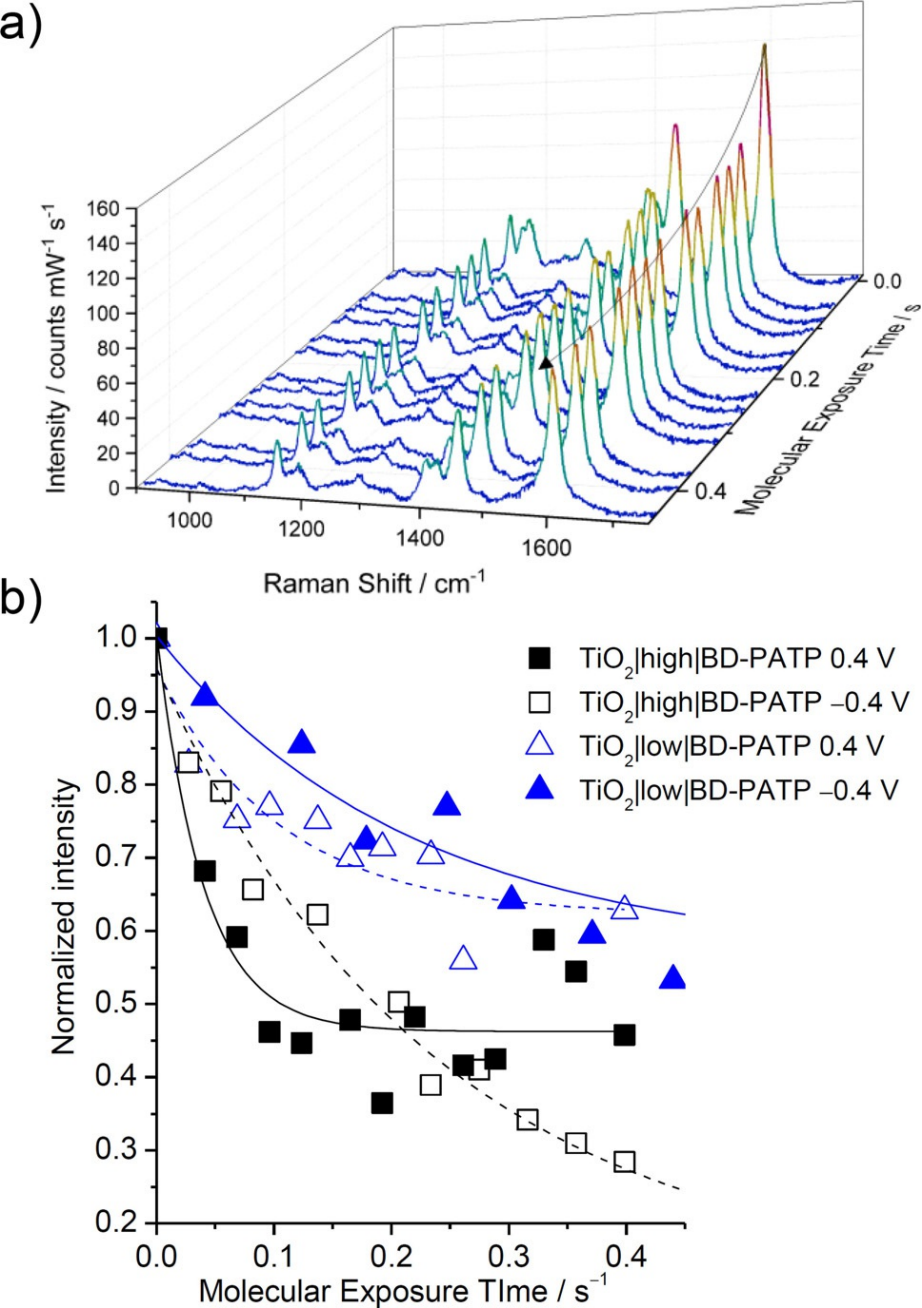
a) Time‐dependent SERR spectra of TiO_2_‐NT|high|BD‐PATP electrodes at open circuit potential in water. The curved arrow indicates the change in intensity of the peak at ≈1599 cm^−1^ over time, which is used to determine the kinetics. *Molecular Exposure Time* denotes the time the laser was irradiating the sample. b) Normalized intensity of the ≈1599 cm^−1^ peak derived from the SERR spectra plotted against molecular exposure time for the different TiO_2_‐NT‐dye systems at different applied potentials. An exponential fit (black and blue lines) was used to determine the decay rate constants. Data derived from SERR spectra recorded on the respective electrodes. Conditions: 10 s accumulation time, average of 10 spectra; laser excitation: 1 mW at 413 nm; sample immersed in 0.1 m phosphate buffered medium, pH 7.

In the employed experimental configuration, the laser excitation served two purposes. On the one hand, it allowed for sensitive detection of the dye molecule on the electrode. On the other hand, the laser concomitantly induced dye degradation by resonantly exciting the molecule.^5^ Upon exposing TiO_2_‐NT|high|BD‐PATP and TiO_2_‐NT|low|BD‐PATP to laser light at 413 nm in aqueous media, a time‐dependent decrease in SERR intensity was observed for both systems as expected for photo‐induced dye degradation (Figure 3 a, Figure S1, Supporting Information).

SERR analysis of the medium after induced photodegradation by drop‐casting of the surrounding water medium on a roughened Ag electrode (Figure S2, details in the Supporting Information) showed bands that rule out the presence of the parent BD‐PATP, indicating that a photo‐induced conversion of BD‐PATP has occurred. The observed bands most likely originate from degradation products (Figure S3). In this respect, a possible degradation product of BD‐PATP is benzidine, which on TiO_2_ may also have converted to higher‐oxidized biphenyl species.^18^

A mere photo‐induced desorption of the dye can be ruled out because in this case BD‐PATP should have been detected in the SERR experiment on the roughened Ag electrode. Due to the immobilization of the dye onto the TiO_2_‐NT surface, the overall dye concentration was too low to perform in‐depth product analysis, which is also beyond the scope of this paper. The capability of TiO_2_‐NT to promote dye‐degradation reactions has already been demonstrated in the literature.^12^

Noteworthy, no decrease in SERR intensity was observed when BD‐PATP on TiO_2_‐NT was exposed to laser light that is off‐resonant with the dye's electronic absorption, that is, at 647 nm (Figure S4). This observation supports that the photodegradation mechanism requires excitation with light of energy sufficient to induce a transition from the ground to the excited state of the immobilized dye. Importantly, both wavelengths are too low in energy to excite the band gap of TiO_2_. Furthermore, exciting BD‐PATP immobilized on roughened Ag electrodes with a 413 nm laser showed no hints for photo‐induced degradation indicating that the TiO_2_ support is required to promote the reaction (Figure S5).

From the time‐dependent decrease of the SERR intensity, photo‐induced degradation kinetics at 413 nm excitation were derived. Note that we report the Molecular Exposure Time (MET) instead of the mere spectral accumulation time as the samples were moved during measurement to avoid thermal degradation (see the Experimental Section for details). Hence, the amount of time each molecule was exposed was significantly less than the actual experimental/spectral accumulation time. The intensity of the most prominent peak at 1599 cm^−1^ assigned to phenyl‐ring stretching vibration 8a/8b mode/s (Wilson notation)^20^ was normalized to the initial intensity, that is, *t=*0 and plotted as a function of MET (Figure 3 b). Here, *t=*0 refers to the initial intensity of the first average spectrum (of 10 s, 10 times) obtained for the dye right at the moment it was exposed to laser. Located 11 cm^−1^ away from the nearest PATP on TiO_2_‐NT peak (1588 cm^−1^), this peak can be considered characteristic for BD‐PATP on TiO_2_‐NT. Fitting an exponential function yielded the apparent decay constants *k*_app_ (=1*τ*_app_) summarized in Table 1.

**Table 1:** Summary of apparent degradation rate constants (*k*_app_) derived from the decrease in intensity of the ≈1599 cm^−1^ peak in the time‐dependent SERR spectra of the dye BD‐PATP on TiO_2_‐NT electrodes at different applied potentials (V vs. Ag/AgCl 3 m KCl).

Electrolyte/solvent	Deionized Water		0.1 m Phosphate Buffer pH 7			
potential [V]			−0.4		+0.4	
electrode type	TiO_2_‐NT|high	TiO_2_‐NT|low	TiO_2_‐NT|high	TiO_2_‐NT|low	TiO_2_‐NT|high	TiO_2_‐NT|low
*k*_app_ [s^−1^] (error)	5.1 (1.4)	3.0 (0.6)	3.6 (0.6)	1.3 (0.4)	25.5 (5)	10.1 (3)
ratio *k*_app_	1.7		2.8		2.5	

It should be noted that the decrease of the 1599 cm^−1^ band reflects the decay of the parent dye‐state over time. Thus, the kinetics we provide should be considered as a convolution of all kinetic processes that in sum lead to irreversible decay of the parent state. Importantly, the kinetics reflect only the relevant interfacial degradation process. On average, the decay rate constant observed for TiO_2_‐NT|high|BD‐PATP (5.1 (1.4) s^−1^) was found to be approximatively 70 % faster than for TiO_2_‐NT|low|BD‐PATP (3.0 (0.6) s^−1^).

To investigate the effects of electrode polarization on the degradation kinetics, potentials were applied to the TiO_2_‐NT electrodes in 0.1 m phosphate‐buffered medium at pH 7. A distinctly different performance of the two employed materials was found. Application of −0.4 V_Ag/AgCl_ revealed rate constants for both systems comparable to those values obtained in water at open‐circuit potential (OCP) before. Also, in this case TiO_2_‐NT|high|BD‐PATP (3.6 s^−1^) exhibited higher rates, which were averagely 2.8 times higher than those found for TiO_2_‐NT|low|BD‐PATP (1.3 s^−1^). However, a desorption of the dye at −0.4 V_Ag/AgCl_ was noted in the absence of laser irradiation. This effect was observed for both TiO_2_‐NT|high|BD‐PATP and TiO_2_‐NT|low|BD‐PATP systems and was studied in a separate experiment on TiO_2_‐NT|high|BD‐PATP in more detail (Supporting Information, Section 2 and Figure S6). The potential‐induced desorption was found to scale with the time keeping the TiO_2_‐electrodes at negative bias, thus, pointing to a role of the cathodic potential in promoting desorption. As such, *k*_app_ values determined at −0.4 V_Ag/AgCl_ are probably a convolution of desorption and degradation kinetics.

In contrast, no desorption effects were observed at 0.4 V_Ag/AgCl_ but a significant increase of the rate constant was noted compared with OCP conditions. Again, for TiO_2_‐NT|high|BD‐PATP (25.5 (5) s^−1^) a faster rate constant by a factor of about 2.5 was observed.

The presented degradation rate constants are by several orders of magnitude higher than values typically reported in the literature for dye degradation mediated by TiO_2_ nanostructures (usually in the order of 10^−8^ to 10^−2^ s^−1^) (selection presented in Table S1, Supporting Information).^15^ This difference, however, is likely related to the fact that different systems are compared compromising a fair assessment. In fact, usually employed experimental conditions involve TiO_2_ suspensions, a soluble dye, and light irradiation at various powers. In most cases, the kinetics were derived by measuring the dye concentration in solution as a function of time. Although such configuration is closer to a real application, the derived reaction kinetics will differ and also reflect slow diffusional processes, for example, of the dye to or of the product away from the electrode. This being said, the TiO_2_‐NT electrodes presented here surpass directly comparable systems, that is, systems in which heterogeneous TiO_2_ nanostructured supports, laser light as excitation source, and an immobilized dye were used. Toumazatou et al. reported degradation of methylene blue immobilized on photonically active inverse‐opal TiO_2_ structures under constant 514 nm laser irradiation with a rate constant of 0.112 min^−1^, which is significantly lower than the reported value here even in the absence of applied potential and in aqueous conditions.^9^ Latter is important because it is expected that the absence of solvent will also promote thermally induced degradations. To the best of our knowledge, no other system exists that would allow for a fair comparison.

The observed overall high capability of the presented TiO_2_‐NT electrodes to promote photo‐induced degradation can be attributed to their optical properties. As a matter of fact, improved photocatalytic activity through photonic structures found for TiO_2_‐NT systems has been demonstrated in the literature albeit not with this magnitude.^7^, ^9^ The higher performance of TiO_2_‐NT|high over TiO_2_‐NT|low, under all measured conditions, points to this material's specific superior optical properties. This can be understood by considering the stronger electromagnetic field enhancement provided by the TiO_2_|high electrode as recently shown by us.^13^ In our previous work, Raman signal enhancement of probe molecules at the TiO_2_‐NT interface was used to investigate the field enhancement. An approximately 7 times higher Raman signal enhancement on TiO_2_|high than on TiO_2_|low was noted. This translates to a 2.6 times higher electromagnetic field strength (square root of 7) and, in fact, matches nicely the factor of rate‐constant increase found for TiO_2_‐NT|high under all measured conditions (Table 1). Thus, the results indicate a direct correlation between the electromagnetic‐field enhancement of a support and its photo‐induced degradation capability of nanostructures. This establishes in fact a facile and elegant way to assess a material's capability to promote interfacial degradation reactions through its Raman enhancement factor.

The observed potential dependence of the degradation rate constants suggests an electronic influence. In this context, it has already been shown that applying a negative bias results in filling‐up acceptors states in TiO_2_ hindering the electron injection and enhancing recombination processes.^21^ Moreover, it could potentially favor breaking of the Ti−S bonds and hence lead to (reductive) desorption of the dye as a competing side reaction. Such behavior has been observed for thiol‐bound compounds on metal electrodes.^22^ Importantly, an overall decreased photocatalytic degradation activity should be noted in this case. Both are in fact observed in our experiments if we consider the rate constants derived at −0.4 V_Ag/AgCl_ as a rough estimation (convolution of desorption and degradation) and compare them to those obtained at 0.4 V_Ag/AgCl_ (Table 1). Conversely, applying +0.4 V could accelerate the degradation as a result of potentially three effects. First, electron‐injection kinetics could be enhanced due to the additional driving force as well as the availability of more unoccupied acceptor states.^21^ Second, the positive potential could assist in transporting the injected electron into the bulk away from the oxidized dye, which would enhance charge separation efficiency. Finally, the positive potential could afford increased adsorption of negatively charged PO_4_^2−^ ions along with removal of K^+^ ions at the TiO_2_‐NT interface. This corresponds to the formation of a more negatively charged Helmholtz layer resulting in an interfacial electric field pointing to the interface. This electric field will enhance injection kinetics as well as decrease recombination rates as also discussed for cation/electric field dependence in DSSCs.^23^

## Conclusions

We presented the application of TiO_2_ nanotube electrodes for hosting the photocatalytic degradation of the azo‐dye BD‐PATP. The extraordinary optical properties of the presented TiO_2_ nanotube system afforded highest degradation rates in the s^−1^ region under direct laser illumination in aqueous media. The degradation activity was found to be directly linked to the Raman enhancement factor and therefore attributed to the material's ability to accommodate enhanced electromagnetic fields within its nanostructure. The performance could be furthermore significantly increased by applying a positive bias. Our study demonstrates the benefit of applying surface‐enhanced Raman spectroscopy to investigate interfacial dye degradation on photonic materials focusing thereby only on the important interfacial reactions. Moreover, the approach allows for readily assessing any material's photo‐induced degradation capability at its surface through the afforded Raman enhancement factor. The derived knowledge is of particular importance for rationally designing and developing novel highly active photocatalysts. Further studies will focus on the activity of the TiO_2_ nanotube electrodes at other laser excitation wavelengths as well as towards other environmentally relevant dyes.

## Experimental Section

**Sample preparation**: The main procedure for the preparation of the sample for SERR measurement is given in Figure 1. The formation of TiO_2_ by anodization is given in the literature.^24^ Briefly, a potential of 20 V (Hameg, HMP2020; with Ag/AgCl, 3 m KCl, leak‐free as reference and Pt sheet as counter electrode) was applied on Ti foil (99.6 %, Chempur) in the presence of 0.202 m NH_4_F (99.99 %, Sigma–Aldrich) solution in 1:1 glycerol (≥99.5 %, Sigma–Aldrich)/water for 2 h. After anodization, the electrode was rinsed with copious amounts of deionized water and dried under nitrogen stream. The TiO_2_‐NT substrates were then annealed at 300 (TiO_2_‐NT|low) or 475 °C (TiO_2_‐NT|high) for 2 h in a Nabertherm oven to improve its crystallinity. The ramp‐up was done at 15 °C min^−1^ until the desired temperature. *p*‐aminothiophenol, PATP (97 %, Sigma–Aldrich), was then deposited on TiO_2_‐NT by immersing the electrode in 2 mm ethanolic (Analytical Grade, 99.9 %, Fischer Scientific Company, Germany) solution of the thiol and allowing self‐assembled monolayers (SAMs) to form. Deionized water (resistance >18 MΩ, Millipore, Eschborn, Germany) was used for washing and solution preparation.

A simple diazotization reaction adapted from Han et al.^16^ was then conducted in an ice bath to yield benzidine‐*p*‐aminothiophenol, BD‐PATP from PATP on TiO_2_‐NT. Briefly, fresh 5 % sodium nitrite (99.999 %, Sigma–Aldrich) solution was prepared and, together with 0.1 m hydrochloric acid solution (ACS reagent, 37 %, Sigma–Aldrich), added into the vial containing the TiO_2_‐NT electrode. After 10 min, the solution was removed and aqueous benzidine (≥98 %, Sigma–Aldrich) solution (≥2 mm) was added into the TiO_2_‐NT electrode. The reaction was stopped after 10 min by harvesting the functionalized electrode, washing it with copious amounts of water, and gently drying with a stream of nitrogen gas. This sample was then used for SERR measurements.

**SER/SERR measurement**: The functionalized TiO_2_ NT electrodes were assembled in a spectro‐electrochemical cell (Pt wire counter and Ag/AgCl, 3 m KCl reference electrodes) and ≈4 mL of water or 0.1 m phosphate (from K_2_HPO_4_ and KH_2_PO_4_ (≥99 %, Merck)), pH 7.0, buffer solution was added. The liquid serves as a heat sink to prevent thermal effects in the degradation procedure during laser exposure. In addition, thermal degradation was prevented by rotating the sample using an XY stage (OWIS GmbH, Germany). To prevent defocusing or shifting of position during measurement, a custom‐built holder provides mechanical stability of the cell during rotation. SER/SERR measurements were performed using Kr^+^ laser at a 413 nm excitation (Coherent Innova 300c) coupled to a confocal Raman spectrometer (Jobin–Yvon, LabRam 800 HR) with a back‐illuminated CCD detector cooled by liquid N_2_. The laser was aligned and then focused on the sample using a Nikon 20× objective (NA=0.35, WD=20 mm) at a laser power of ≈1 mW. The spectral resolution was about 1.2 cm^−1^. Spectra were calibrated with respect to mercury lines and the Raman spectrum of toluene.

Time‐dependent SERR measurements were performed for the degradation experiments. This entailed recording the SERR spectra in intervals of ≈100 s (as an average of 10 spectra, with each spectrum accumulated for 10 s) for a duration of at least 30 min experimental time. In potential‐dependent measurements, a potentiostat (MetrOhm) was used to apply an external potential of +0.4 or −0.4 V (vs. Ag/AgCl, 3 m KCl).

Measurement at off‐resonance was performed at 647 nm using a confocal Raman spectrometer (LabRam HR‐800 Jobin Yvon) equipped with liquid N_2_‐cooled CCD camera (Symphony II Horiba). Spectral analysis for background subtraction included polynomial function of the spectra. Further intensity analysis was performed using Origin.

**Calculation of the** ***Molecular Exposure Time*** **(MET)**: The exposure time axis was expressed as *Molecular Exposure Time* (MET) to account for the the mechanical rotation of the electrode during measurements, thereby resulting to intermittent illumination of the sample at each traversed spot. Electrode rotation was employed to prevent thermal degradation as a result of prolonged laser exposure and to allow the molecules in the sample spot to recover thermally or prevent heating up significantly. This was done by constantly moving the sample to yield a circle with a radius, *r*=1 mm in this case, that is illuminated by the laser. Considering that, at a time, only a fraction of the circumference was actually exposed to laser (Figure S7, Supporting Information), and that this fraction is approximately equal to the laser spot diameter, *D*:^25^

(1) D=(1.22λ)/(N.A.)

in which *λ* and N.A. refer to the laser‐line excitation wavelength (i.e., 413 nm) and numerical aperture (i.e., 0.35), respectively, one can convert the apparent experimental measurement time, *t*_exp_, to give the time in which each molecule was actually exposed to laser by expressing them in ratios of the distance and time of the laser path with respect to the circumference so that:

(2) $$Molecular\;Exposure\;Time=t{}_{\exp}/2\pi r\times 1.22\;\lambda /N.A.$$

The value 1.22 is a diffraction‐pattern limitation factor.

**Determination of apparent rate constant** ***k***_**app**_: After obtaining the SERR spectra, the intensities of the most prominent peak (1599 cm^−1^) were divided by the initial intensity (*t=*0) to express the peaks in values ≥1 and easily see the succeeding amounts as fractions of the initial intensity. The values were not normalized from 0 to 1 because the dye was still not fully degraded within the set experimental time (≈30 min experimental time per measurement). The intensities of this peak (≈1599 cm^−1^) were then plotted against MET. A fit of an exponential decay function afforded the decay constant.

## Conflict of interest

The authors declare no conflict of interest.

## Supporting information

As a service to our authors and readers, this journal provides supporting information supplied by the authors. Such materials are peer reviewed and may be re‐organized for online delivery, but are not copy‐edited or typeset. Technical support issues arising from supporting information (other than missing files) should be addressed to the authors.

Supplementary
