# Supplementary material for: Accelerated Photo‐Induced Degradation of Benzidine‐p‐Aminothiophenolate Immobilized at Light‐Enhancing TiO2 Nanotube Electrodes
Source: Chemistry. 2019 Nov 18;25(70):16048–53. doi: 10.1002/chem.201902963 (PMC6972621; doi:10.1002/chem.201902963)
Supplement: Supplementary file 1 — Supplementary [file CHEM-25-16048-s001.pdf]

CHEMISTRY
A European Journal

Supporting Information

Accelerated Photo-Induced Degradation of Benzidine-*p*-Aminothiophenolate Immobilized at Light-Enhancing TiO_2 Nanotube Electrodes

Christine Joy Querebillo,[a, b, c] Ibrahim Halil Öner,[a] Peter Hildebrandt,[b] Khoa Hoang Ly,*[a] and Inez M. Weidinger*[a]

chem_201902963_sm_miscellaneous_information.pdf

SUPPLEMENTARY INFORMATION

Table of content

1. Degradation study 2

2. Desorption study 2

3. Supporting Figures and Tables 4

4. Supplementary references 14

1. Degradation study

Degradation in water (no potential applied). To characterize the product/s after photoinduced BD-PATP degradation, few adjustments to the SERR measurement setup have been incorporated to increase the final concentration of degraded species in the water medium. Specifically, the laser was moved over an increased electrode surface area corresponding to at least ~18 times of the original area used for degradation measurements (Fig. S2). Following this, the water medium (4 mL) surrounding the electrode was collected and drop-casted onto a rough silver disk for SERR measurement to detect the generated species.

The roughening of the silver disk was achieved by following published procedures.[1] Briefly, silver disk was polished with 3M polishing films, washed with copious amounts of deionized water, and subjected to oxidation-reduction cycles in 0.1 M KCl to create a nano-rough surface. The SERR spectrum of the blank roughened silver was recorded prior to drop casting for reference. The drop-casted water/solution containing degraded molecules was allowed to dry and the disk was subjected to SERR measurement at 413 nm laser excitation to give the spectrum for degradation products. Fig. S3 shows the SERR difference spectrum of the degradation products (red), *i.e.*, the spectrum of the blank roughened silver was already subtracted plotted against the spectrum of the dye immobilized on roughened Ag (blue) for reference. Here, one clearly sees the difference between the degradation product/s from the original dye indicating that degradation (and not mere desorption) occurred.

2. Desorption study

Desorption at –0.4 V in 0.1 M potassium phosphate buffer, pH 7. To assess desorption effects, the sample was assembled in the same electrochemical cell as for the degradation experiments, immersed in 0.1 M phosphate buffered medium, pH 7, and the cell was attached to the potentiostat (nano- or micro-autolab). An external potential of –0.4 or 0.4 $V_{Ag/AgCl}$ was applied, and the first SERR spectrum was accumulated. After this, the laser was switched off but the potential bias was maintained. After ≥ 30 min experimental time, SERR spectra were recorded after every 100 s (or 10 s times 10 accumulation for obtaining the average spectra) with continuous laser irradiation.

The resulting plot is presented in Fig. S6 showing the electrochemical desorption of the dye from TiO_2-NT when a potential of –0.4 $V_{Ag/AgCl}$ was applied for ca. 50 min or more of the experimental time. Between the initial SERR spectrum and the 2nd spectrum, the laser was switched off for ~35 min (equivalent to ~0.475 s *Molecular Exposure Time*), while the negative potential was continuously applied to the electrode. Afterwards, the 2nd spectrum was obtained showing only a normalized intensity of ~50 %. This value is substantially lower than what is expected for the second data point for which the laser illumination time required to obtain the SERR spectrum should result in a signal with about ~80 % of the normalized intensity (Fig S6, 3rd data point). This means that ~30 % of the

intensity decay after ~35 min is due to the application of negative potential, which is attributed to desorption process occurring at this potential.

No desorption effects were observed in the absence of an applied potential or at 0.4 $V_{Ag/AgCl}$. In those cases, when the laser focus was transferred to a fresh unilluminated spot on the TiO_2-NT electrode, the SERR intensity of BD-PATP in the new spot was found to be similar to the initial intensity in the first recorded spot. Hence, at 0.4 V or when no potential is applied, desorption is insignificant or minimal such that the apparent rate constants can be expected to be dominated by laser-induced degradation reactions.

3. Supporting Figures and Tables

Title	Page No.
**Figure S1**. Time-dependent SERR spectra of BD-PATP on TiO_2-NT|low electrode.	5
**Figure S2.** Scheme for the TiO_2-NT|BD-PATP degradation study.	5
**Figure S3**. 413 nm excited SERR spectra of the degradation products drop cast on rough Ag.	6
**Figure S4**. SER spectra of TiO_2-NT|high|BD-PATP excited at 647 nm (Krypton Laser (Sabre Innova Kr)) at the start of continuous laser exposure (t = 0) and after 0.4 s of *Molecular Exposure Time.*	6
**Figure S5.** SERR spectra of BD-PATP on roughened Ag electrodes excited at 413 nm with 1 mW laser power at the start of continuous laser exposure (t = 0) and after 0.4 s of *Molecular Exposure Time*.	7
**Figure S6**. Potential-induced desorption of BD-PATP on TiO_2-NT|high at an applied potential of –0.4 $V_{Ag/AgCl}$ monitored using SERR spectroscopy.	7
**Figure S7**. Sketch of the rotation pattern of the laser illumination used in the time resolved SERR experiments to determine the degradation rate constants.	8
**Table S1**. List of parameters and decay rate constants of photodegradation studies of dye on TiO_2 nanostructures.	9

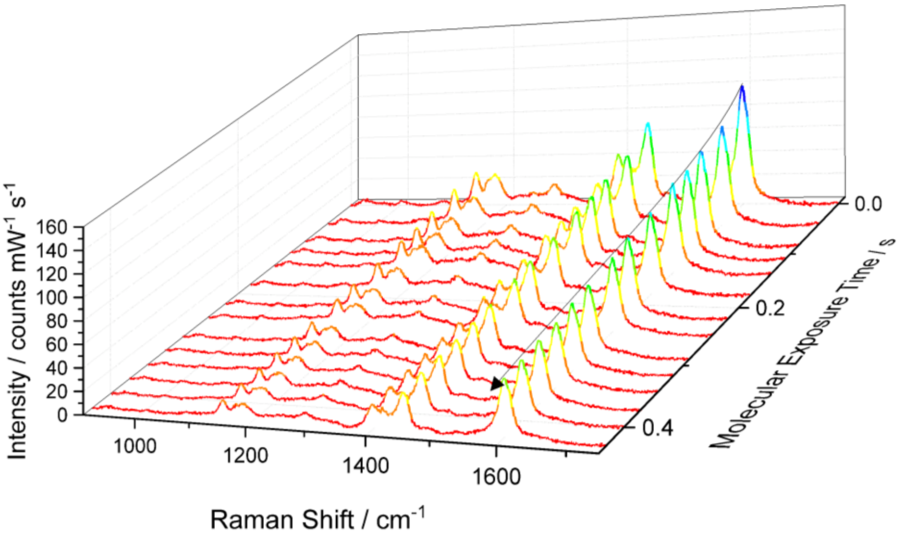

Figure S1. Time-dependent SERR spectra of BD-PATP on TiO_2-NT|low electrode. The curved arrow highlights the change in intensity of the peak at ~1599 cm^{-1} over time. *Molecular Exposure Time* denotes the time the laser was in actual contact with the sample. Conditions: 10 s, average of 10 spectra; laser excitation: 1 mW at 413 nm; sample immersed in deionized water.

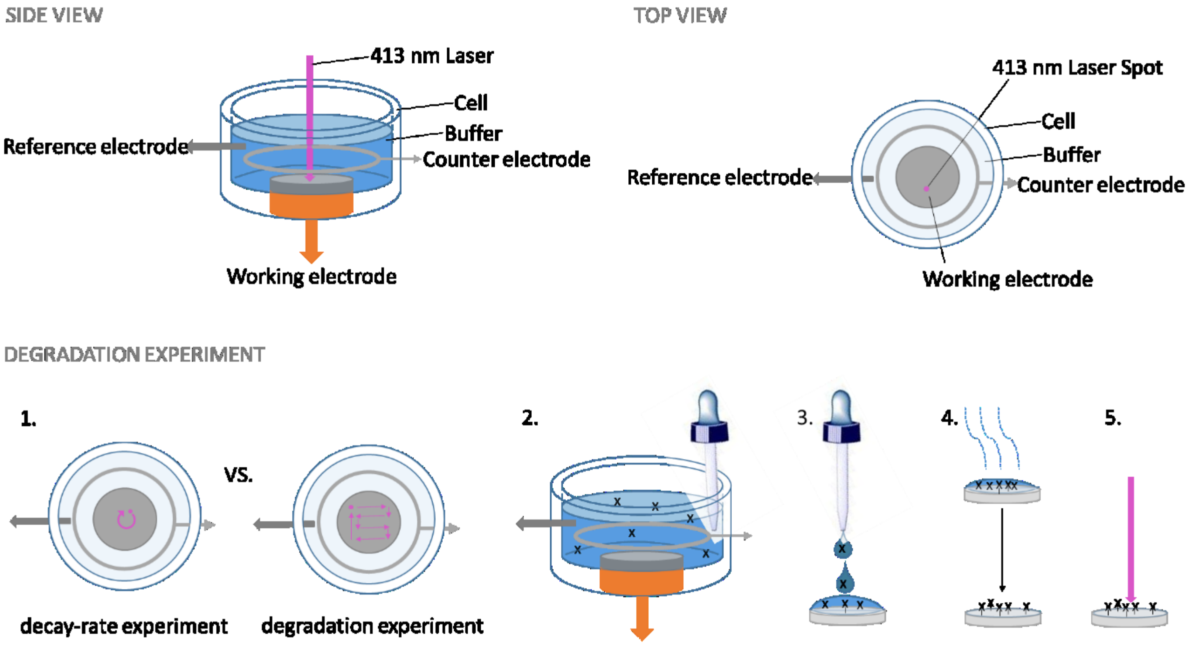

Figure S2. Scheme for the TiO_2-NT|BD-PATP degradation experiments. Above the scheme, the side and top views of the electrochemical setup are shown and labelled. The flow of the experiment is shown starting with a comparison of the laser-path patterns between the experiments for determining the decay constants vs. the degradation experiment (Degradation Experiment, step 1). The pattern adjustment allowed for exposure of a greater surface area of the TiO_2-NT|BD-PATP to increase the amount of degraded species, X. Experiment was done such that the molecules were exposed to a similar *Molecular Exposure Time* as in the decay-rate experiments. The entire volume of the liquid medium was then collected (step 2), drop-casted on rough Ag (step 3), and allowed to evaporate (step 4). The drop-casted roughened Ag was then analyzed by SERR measurement at 413 nm (step 5).

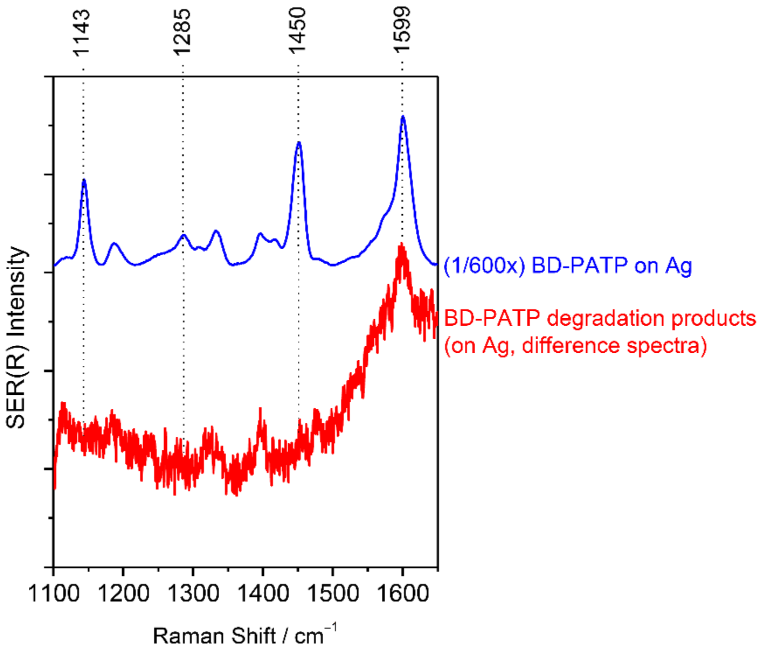

Figure S3. 413 nm excited SERR spectra of the degradation products drop cast on rough Ag (red). Rough Ag was chosen due to the strong plasmonics-based surface-enhancement provided by the support, which enables detection also of low concentrations of compounds. The BD-PATP deposited on roughened Ag (Ag|BD-PATP; in a similar way as the TiO_2-NT|BD-PATP in this study) is shown here (blue) for comparison. The spectrum of the degradation products is distinctly different to the spectrum of the parent BD-PATP indicating a degradation process has taken place.

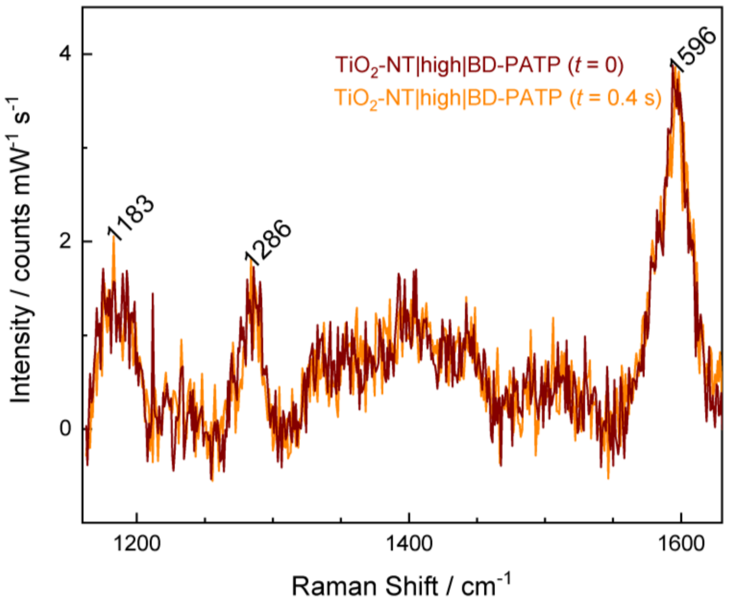

Figure S4. SER spectra of TiO_2-NT|high|BD-PATP excited at 647 nm (Krypton Laser (Sabre Innova Kr)) at the start of continuous laser exposure (t = 0) and after 0.4 s of *Molecular Exposure Time*. Despite the lower spectral quality due to off-resonance Raman measurements, no decrease in intensity after 0.4 s was observed, indicating that the photodegradation of BD-PATP on TiO_2-NT requires energy ≤ of 413 nm (but > 647 nm). Conditions: 10 s, average of 10 spectra; laser power of 1 mW; sample immersed in deionized water.

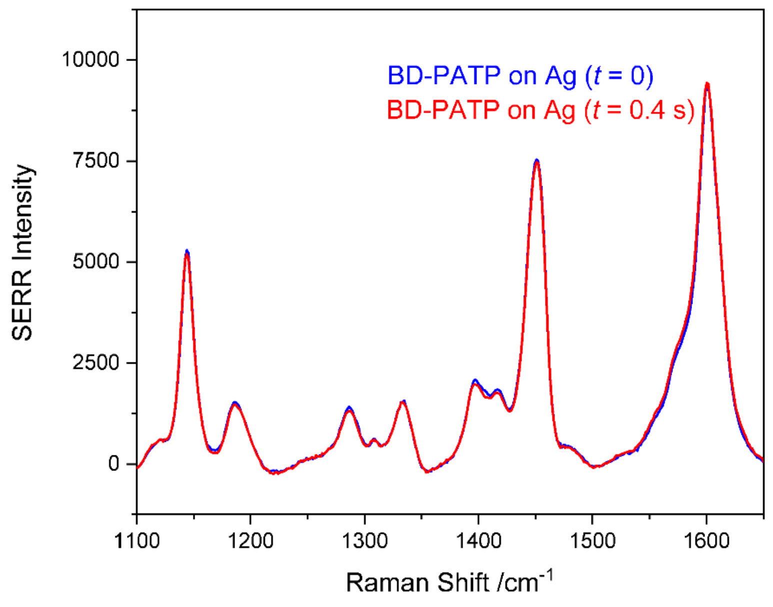

Figure S5. SERR spectra of BD-PATP on roughened Ag electrodes excited at 413 nm with 1 mW laser power at the start of continuous laser exposure (t = 0) and after 0.4 s of *Molecular Exposure Time*. No spectral difference is observed indicating that no photo-induced degradation occurs on Ag under the applied conditions.

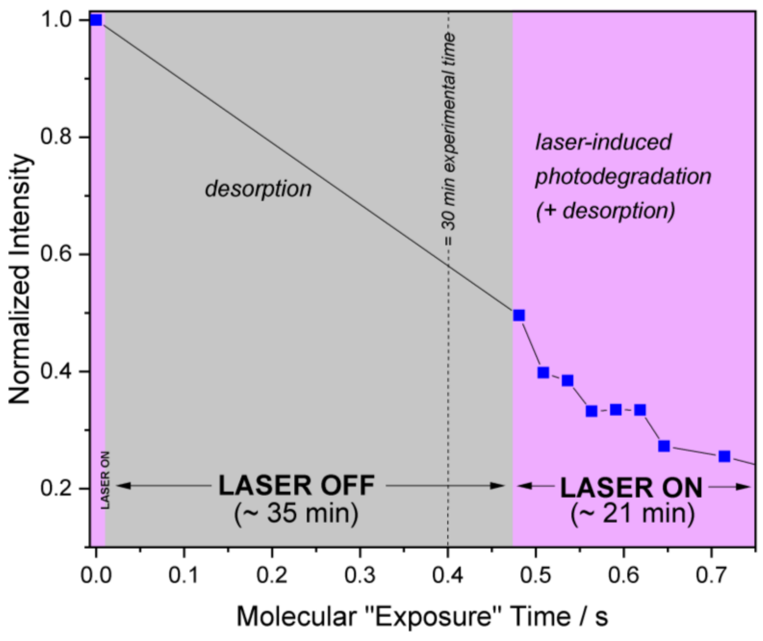

Figure S6. Potential-induced desorption of BD-PATP on TiO_2-NT|high at an applied potential of –0.4 $V_{Ag/AgCl}$ monitored using SERR spectroscopy. BD-PATP was deposited on TiO_2-NT|high and a potential of –0.4 V vs. Ag/AgCl continuously applied. After the initial SERR spectra, the laser (413 nm) was switched off to prevent spectral decay due to photodegradation and induce a decay mainly due to electrochemical desorption. After around 35 min (or equivalent to 0.475 s *Molecular Exposure Time*), the laser was again switched on for SERR measurement.

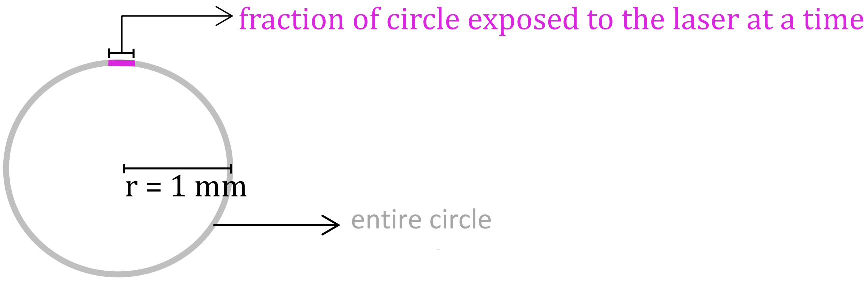

Figure S7. Sketch of the rotation pattern of the laser illumination used in the time resolved SERR experiments to determine the degradation rate constants.

Table S1. List of parameters and decay rate constants of photodegradation studies of dye on TiO_2 nanostructures.

Light source Type	Wave-length	Power	System	Molecule/Reaction	*K* Reported/Estimated	*k* (s^{-1})	Detection	Ref
125 W medium pressure mercury lamp (Philips)	Most likely UV	125 W	Dye derivative in aqueous TiO_2 suspension	Photocat degradation of dye derivative	~ 0.03 min^{-1} (graph estimate)	~5.2-6.2 $\cdot 10^{-4}$	Concentration change, UV vis (625 nm peak)	[2]
125 W medium pressure mercury lamp (Philips)	Most likely UV	125 W	Dye derivative in aqueous TiO_2 suspension	Photocat degradation of dye derivative	~0.046 min^{-1} (graph estimate)	~6.6-7.6 $\cdot 10^{-4}$	Concentration change, UV vis (638 nm peak)	[2]
UV light irradiation using 125 W high-pressure mercury lamp (Philips, HPLN, emission bands in the UV region at 304, 314, 335 and 366 nm, with maximum emission at 366 nm)	UV	125 W	TiO_2 (hydrothermally produced and commercial) as catalyt for UV-assisted dye degradation. UV lamp was located at 10 cm above the solution	UV assisted degradation of methylene blue in aqueous solution	0.18-0.70 10^{-2} min^{-1}	0.3-1.17 $\cdot 10^{-4}$	Concentration change det. By visible spectroscopy (675 nm)	[3]
same	UV		TiO_2/C composite as catalyt for UV-assisted dye degradation UV lamp was located at 10 cm above the solution	UV assisted degradation of methylene blue in aqueous solution	0.31-1.02 10^{-2} min^{-1}	0.517-1.7 $\cdot 10^{-4}$	Concentration change det. By visible spectroscopy (675 nm)	[3]
150 W tungsten halogen lamp with glass optical filter)	Vis (> 420 nm)	150 W	TiO_2/C composite as catalyt for dye degradation	degradation of methylene blue in aqueous solution	5.832 10^{-2} min^{-1} (graph estimate)	9.72 $\cdot 10^{-4}$	Concentration change det. By visible spectroscopy (675 nm)	[3]
UV low P mercury lamp (few emission lines mainly at 264 & 365 nm)	254 nm	Intensity of 2.7 9 10-4 W/cm^2	Anatase nanowire (entangled)	UV photocatalytic degradation of venlafaxine	0.0375 min^{-1}	6.25 $\cdot 10^{-4}$	Solid phase microextraction (SPME) and HPLC–MS/MS to determine the concentration in the solution	[4]

Light source Type	Wave-length	Power	System	Molecule/Reaction	*k* Reported/Estimated	*k* (s^{-1})	Detection	Ref
UV low P mercury lamp (few emission lines mainly at 264 & 365 nm)	254 nm		Rutile nanowire (aligned)	UV photocatalytic degradation of fluoxetine	0.2 min^{-1}	$3.33 \cdot 10^{-3}$	Solid phase microextraction (SPME) and HPLC–MS/MS to determine the concentration in the solution	[4]
Mercury lamp	UV	Irradiation density with mercury lamp 10^{17} quant/cm^2 (ours, my estimate: 2 x 10^18 quant/cm^2)	Dye on mesoporous TiO_2 film (template sol-gel synthesis)films (air dried)	Photodegradation of dye acridine yellow on the surface of mesoporous TiO_2 film (template sol-gel synthesis)	$1.5 \cdot 10^{-1}$ min^{-1}	$2.5 \cdot 10^{-3}$	Time-dependent changes in absorption spectra (~445 nm)	[5]
Laser (50 x, 0.5 NA) (dia=1 µum)	785, 633, and 532 nm	0.5 mW	CV dyes on NaYF4:Yb,Tm@TiO_2/Ag (a NIR photoactive catalyst) (hexagonal microcrystal core + TiO2/Ag comby shell)	Photocatalytic degradation of CV dye	0.02612 s^{-1} (785 nm), 0.00715 s^{-1} (633 nm), 0.00565 s^{-1} (532 nm) (estimation)		Time-dependent SERS	[6]
Laser (50 x, 0.5 NA) (dia=1 µum)	785 nm	0.5 mW	CV dyes on TiO_2TiO2/Ag (comby TiO_2TiO2/Ag shell)	Photocatalytic degradation of CV dye	0.00046 s^{-1}		Time-dependent SERS	[6]
Low P Hg vapour lamp	254 nm	6x8 W (254 nm) (surrounding the reactor)	Aqueous solution of dye with Degussa P25 nanoparticles	Photocatalytic degradation of dye reactive Red 2	0.0101-0.0680 min^{-1}	1.683-11.33 $\cdot 10^{-4}$	Concentration change monitored with a spectrophotometer	[7]

Light source Type	Wave-length	Power	System	Molecule/Reaction	*k* Reported/Estimated	*k* (s^{-1})	Detection	Ref
Hg arc lamp (Oriel) equipped with a water-cooled IR filter	<350 nm	100 W	Epitaxial TiO_2 film in dye solution	Photocatalytic decomposition of an organic dye (methyl orange)	3.25 E-3 /min (anatase)<br>1.75 E-3 /min (rutile)	2.92-5.42 $\cdot 10^{-5}$	methyl orange concentration versus reaction time for a TiO_2 film (UV vis spectroscopy)	[8]
UV-LED	365 nm (peak emission wavelength)	450 mW (1,9 mW/cm^2 reached the foil)	Titanium nanotube array in an aqueous solution of beta-blocker metropolol	Photocatalytic degradation of meteropolol	0.0086-0.0206 min^{-1}	1.43-3.85 $\cdot 10^{-4}$	LC-MS/MS analysis	[9]
Pulsed laser (Spectra Physics Nd: YAG laser (Model GCR 250), with 8nsec pulse width)	355 nm		TiO_2 powder (commercially available, Fischer Scientific Co. USA) in water with phenol	Photocatalytic degradation of phenol in water with TiO_2 powder	0.0081 min^{-1}	$1.35 \cdot 10^{-4}$	Change in concentration with UV vis	[10]
UV lamp (OsramUltra Vitalux)	UV	300 W	TiO_2 suspension (prepared by sol-gel technique) in water	Photodegradation of 2-chlorophenol	0.0189 min^{-1}	$3.15 \cdot 10^{-4}$	analyzed by means of UV spectrophotometry at a wavelength of 275 nm	[11]
Simulated sunlight (Xenon lamp, Newport Corporation, Irvine, CA, USA)	200-20000 nm	300 W	TiO_2 nanosheets prepared via a hydrothermal reaction	Degradation of methylene in aqueous solution with TiO_2 nanosheets (or doped TiO_2 nanosheets)	0.04568 min^{-1} (TiO_2, sunlight sim)<br><br>0.2402 min^{-1} (TiO_2 S, sunlight sim)	$7.6 \cdot 10^{-4}$<br><br>$4.6 \cdot 10^{-3}$	UV vis at 664 nm	[12]
Visible light (same as above, modified with mirrors)	420–630 nm	300 W	TiO_2 nanosheets prepared via a hydrothermal reaction	Degradation of methylene in aqueous solution with TiO_2 nanosheets (or doped TiO_2 nanosheets)	0.02119 min^{-1} (TiO_2, vis, linear fit)<br><br>0.09215 (TiO_2-s, vis)	$3.53 \cdot 10^{-4}$<br><br>$1.54 \cdot 10^{-3}$	UV vis at 664 nm	[12]

Light source Type	Wave-length	Power	System	Molecule/Reaction	*k* Reported/Estimated	*k* (s^{-1})	Detection	Ref
UV light (high-pressure Hg vapor lamp (Philips, India))	Mainly 365 nm	125 W	Combustion-synthesized TiO_2 nanoparticles in aq. solution of dye	Degradation of rhodamine 6g using combustion synthesized TiO_2	0.3333 min^{-1}	$5.55 \cdot 10^{-3}$	Uv vis at 525 nm	[13]
Visible light (350 W Xe lamp equipped with a filter so that wavelength is > 400 nm)	>400 nm	350 W	Methylene blue ($1*10^{-5}$ mol/L) in solution degraded by TiO_2 np or inverse opal (IO) photocatalysts	Degradation of methylene blue with TiO_2 nps and IO	6 E-3/min (TiO_2 np) (linear fit, graph estimate),<br>26.44 E-3/min (TiO_2 IO, graph estimate),<br>41.66 E-3/min (TiO_2 IO doped with N, sensitized with PANI and with 240 nm PS template, graph estimate)	$1 \cdot 10^{-4}$ (TiO_2 np),<br>$4.41 \cdot 10^{-4}$ (TiO_2-IO),<br>$6.94 \cdot 10^{-4}$ (P-NTiO_2 IO)	UV vis at ca. 660 nm (MB peak)	[14]
Obtained from a solar simulator (1000 W xenon lamp with AM 1.5G filter) and optical filters	495-700 nm	1000 W; light intensity adjusted to 100 mW cm^{-2}	Photocatalyst sample placed in a 0.5 µM MB solution	degradation of methylene blue (MB) under visible-light illumination in the presence of photocatalyst	0.028 min^{-1} (bare mesoporous-TiO_2 film)<br>0.134 min^{-1} (low C TiO_2 IO)<br>0.220 min^{-1} (high C TiO_2 IO)	$4.67 \cdot 10^{-4}$ (TiO_2 meso),<br>$2.23 \cdot 10^{-3}$ (low C-TiO_2 IO),<br>$3.67 \cdot 10^{-3}$ (high C-TiO_2 IO)	Optical absorbance of MB (60-665 nm)	[15]

Light source Type	Wave-length	Power	System	Molecule/Reaction	*k* Reported/Estimated	*k* (s^{-1})	Detection	Ref
365 nm source was a set of five Philips TL K05 UVA lamps, 254 nm source was a Philips TUV PL-S lamp at a distance of 10 cm from the quartz cell.	365 nm and 254 nm	Irradiance of on top of the solutions of 57 W m^2 (for 365 nm)	Most probably, photocatalyst powder immersed in phenol solution	Degradation of phenol<br><br>(Note: Absolute rates are not directly comparable between the two experiments, also because of the different scattering of light at the two wavelengths)	0.89959 min^{-1} (Degussa, 254 nm, graph estimate),<br><br>0.30961 min^{-1} (IO, 254 nm, graph estimate),<br><br>0.14033 min^{-1} (Degussa, 365 nm, graph estimate),<br><br>0.06378 min^{-1} (Degussa, 365 nm, graph estimate)	$1.5 \cdot 10^{-2}$ (Degussa, 254 nm),<br><br>$5.16 \cdot 10^{-3}$ (Inverse Opal, 254 nm),<br><br>$2.34 \cdot 10^{-3}$ (Degussa, 365 nm),<br><br>$1.1 \cdot 10^{-3}$<br><br>(Degussa, 365 nm, graph estimate)	Disappearance of phenol monitored by HPLC system and followed at 210 nm.	[16]

4. Supplementary references

[1] H. Wackerbarth, U. Klar, W. Günther, P. Hildebrandt, *Appl. Spectrosc.* **1999**, *53*, 283–291.

[2] M. Saquib, M. Faisal, M. Abu Tariq, M. Muneer, *Adv. Sci. Lett.* **2010**, *3*, 512–517.

[3] M. Maletić, M. Vukčević, A. Kalijadis, I. Janković-Častvan, A. Dapčević, Z. Laušević, M. Laušević, *Arab. J. Chem.* **2016**, DOI 10.1016/j.arabjc.2016.06.020.

[4] A. Hu, X. Zhang, D. Luong, K. D. Oakes, M. R. Servos, R. Liang, S. Kurdi, P. Peng, Y. Zhou, *Waste and Biomass Valorization* **2012**, *3*, 443–449.

[5] N. P. Smirnova, N. I. Surovtseva, T. V. Fesenko, E. M. Demianenko, A. G. Grebenyuk, A. M. Eremenko, *J. Nanostructure Chem.* **2015**, *5*, 333–346.

[6] Y. Ma, H. Liu, Z. Han, L. Yang, J. Liu, *J. Mater. Chem. A* **2015**, *3*, 14642–14650.

[7] M. V. Shankar, B. Neppolian, S. Sakthivel, B. Arabindoo, M. Palanichamy, V. Murugesan, *Indian J. Eng. Mater. Sci.* **2001**, *8*, 104–109.

[8] T. Luttrell, S. Halpegamage, J. Tao, A. Kramer, E. Sutter, M. Batzill, *Sci. Rep.* **2015**, *4*, 1–8.

[9] Y. Ye, Y. Feng, H. Bruning, D. Yntema, H. H. M. Rijnaarts, *Appl. Catal. B Environ.* **2018**, *220*, 171–181.

[10] M. A. Gondal, M. N. Sayeed, A. Alarfaj, *Chem. Phys. Lett.* **2007**, *445*, 325–330.

[11] G. V Morales, E. L. Sham, R. Cornejo, M. E. F. Torres, *Lat. Am. Appl. Res.* **2013**, *43*, 325–328.

[12] W. Wang, Z. Wang, J. Liu, Z. Luo, S. L. Suib, P. He, G. Ding, Z. Zhang, L. Sun, *Sci. Rep.* **2017**, *7*, 46610.

[13] T. Aarthi, G. Madras, *Ind. Eng. Chem. Res.* **2007**, *46*, 7–14.

[14] L. Yuan, Z. Yu, C. Li, J. Li, Y. Shen, H. Zhang, C. Wang, A. Xie, *J. Electrochem. Soc.* **2014**, *161*, 332–336.

[15] S. Lee, Y. Lee, D. H. Kim, J. H. Moon, *ACS Appl. Mater. Interfaces* **2013**, *5*, 12526–12532.

[16] F. Sordello, C. Duca, V. Maurino, C. Minero, *Chem. Commun.* **2011**, *47*, 6147–6149.
